# Supplementary material for: Association between Organizational Support and Turnover Intention in Nurses: A Systematic Review and Meta-Analysis
Source: Healthcare (Basel). 2024 Jan 23;12(3):291. doi: 10.3390/healthcare12030291 (PMC10855592; doi:10.3390/healthcare12030291)
Supplement: Supplementary file 1 [file healthcare-12-00291-s001.zip › healthcare-2779538-supplementary.pdf]

Supplementary Table S1. Quality of studies included in the systematic review.

	(Sheng et al., 2023)	(Brunetto et al., 2016)	(Abou Hashish, 2017)	(Shacklock et al., 2014)	(Liu et al., 2018)	(Filipova, 2011)	(Bobbio & Manganelli, 2015)	(Galletta et al., 2011)
1. Were the criteria for inclusion in the sample clearly defined?	√	√	√	√	√	√	√	√
2. Were the study subjects and the setting described in detail?	√	√	√	√	√	√	√	√
3. Was the exposure measured in a valid and reliable way?	√	√	√	√	√	√	√	√
4. Were objective, standard criteria used for measurement of the condition?	√	√	√	√	√	√	√	√
5. Were confounding factors identified?			√		√	√		
6. Were strategies to deal with confounding factors stated?			√		√	√		

7. Were the outcomes measured in a valid and reliable way?	√	√	√	√	√	√	√	√
8. Was appropriate statistical analysis used?	√	√	√	√	√	√	√	√
**Total quality**	Moderate	Moderate	Good	Moderate	Good	Good	Moderate	Moderate
